# Oviposition Deterrents from Extracts of *Eryngium foetidum* Against Potato Tuber Moth *Phthorimaea operculella* Zeller (Lepidoptera: Gelechiidae)

**DOI:** 10.3390/insects16020158

**Published:** 2025-02-04

**Authors:** Yanfen Ma, Xinzhou Yang, Mei Wu, Yunjiao Guo, Wenxia Dong, Rui Tang, Chun Xiao

**Affiliations:** 1 Department of Agronomy and Biological Science, Dehong Teacher’s College, Mangshi 678400, China; mayanfen2005@126.com (Y.M.); guoyunjiaodhsz@163.com (Y.G.); 2 The Research Institute of Ethnic Minority Medicine, Dehong Teacher’s College, Mangshi 678400, China; yxz1149@126.com; 3 School of Chinese Material Medica, Yunnan University of Chinese Medicine, Kunming 650500, China; km_wumei@163.com; 4 State Key Laboratory for Conservation and Utilization of Bio-Resources in Yunnan, College of Plant Protection, Yunnan Agricultural University, Kunming 650201, China; dongwenxia@163.com; 5 Centre for Resource Insects and Biotechnology, Institute of Zoology, Guangdong Academy of Sciences, Guangzhou 510260, China

**Keywords:** *Phthorimaea operculella*, *Eryngium foetidum*, oviposition, deterrent, compound analysis, electrophysiology

## Abstract

The potato tuber moth *Phthorimaea operculella* is a serious boring pest of potato. An integrated ecological approach to sustainable pest management is necessary for the control of this species. This study investigated the effects of minced leaves and plant extracts of *Eryngium foetidum* on the oviposition behavior of PTM females. The behavioral regulatory components of PTM females in response to the extracts were determined using chemical analyses and electrophysiological tests. Individual electroantennographic detection (EAD)-active compounds and mixtures were evaluated using oviposition choice bioassays. The results indicate that minced leaves had a deterrent effect on oviposition. The extracts at low dosages had an attraction effect, while high dosages had a repellent effect on the oviposition of PTM adult females. The dominant compounds of the extracts were *trans*-2-dodecenal and *trans*-2-tridecenal and showed EAD activity. *trans*-2-dodecenal, *trans*-2-tridecenal and their mixtures showed significant oviposition-repellent effects toward the PTM. The oviposition stimulation indices (OSIs) of *trans*-2-dodecenal and *trans*-2-tridecenal were −100% and −94.03% at 10 mg/mL, respectively. The OSIs of mixtures at natural ratios of 10 mg/mL and 5 mg/mL were −95.11% and −90.96%, respectively. The results can be used for the further development of ecological control strategies for this pest species.

## Simple Summary

Many phytophagous insects have an outstanding ability to locate their host, showcased by their search for food and oviposition sites using volatiles from plants. Volatile organic compounds (VOCs) derived from non-hosts, especially aromatic plants and medicinal plants, can protect the host crop by masking the host’s odorants and thereby deterring phytophagous pests. This technique has achieved satisfactory results for controlling pests, especially for boring pests. The potato tuber moth (PTM) *Phthorimaea operculella* (Zeller) (Lepidoptera: Gelechiidae) is a serious pest to potato plants. The PTM can bore into potato plants and tubers in fields and storage, and tubers thus lose their edible and commodity value. Ecological methods for the sustainable control of this species are in great demand. This study indicated that minced *Eryngium foetidum* leaves and plant extracts can deter PTM oviposition. The dominant compounds of the plant extracts were *trans*-2-dodecenal and *trans*-2-tridecenal, which elicited antennal responses and had significant oviposition repellent effects against the PTM. The mixture of *trans*-2-dodecenal and *trans*-2-tridecenal at natural ratios also significantly deterred oviposition. This study elucidated how plant volatiles influence and determine the oviposition of PTM females. Our findings provide a reference for developing ecology-based control strategies for this infamous potato pest.

## 1. Introduction

The potato tuber moth (PTM) *Phthorimaea operculella* (Zeller) (Lepidoptera: Gelechiidae) is a serious pest of potato crops (*Solanum tuberosum* L.), as well as other Solanaceous plants, such as tobacco (*Nicotiana tabacum* L.) and tomato (*Solanum lycopersicon* L.), both in the field and in storage [1,2]. The species is widely distributed in warm temperate, tropical, and subtropical regions worldwide [3,4,5]. The PTM can infest potato plants and tubers in fields and storage, with yield losses of up to 100% when infestation occurs during storage [2,6]. Infested potato tubers and leaves present galleries bored by the larvae, and the tubers thus lose commercial value [2,6,7,8]. Most chemical insecticides are ineffective due to the concealed damage caused by larval mining, and the development of resistance to chemical pesticides can harm the environment [9,10].The parthenogenesis of this species reduces the control potential of sex pheromones [11,12]. Thus, the manipulation of oviposition behavior is one of the key control approaches for this species. This makes it necessary to screen for novel oviposition cues that are eco-friendly, safe, and cost-effective.

Many phytophagous insects have a suite of behaviors designed to locate their host plants, often manifested as a search for oviposition sites using plant volatiles [13,14,15]. Even in a complex environment, the insects can discriminate host odorants from non-host volatiles [16,17,18]. Volatile organic compounds (VOCs) derived from non-hosts can protect the host crop by masking the host’s odorants and thereby deter phytophagous insect pests [16,19,20,21]. This technique has achieved satisfactory results in controlling pests, especially boring pests such as *Atherigona orientalis*, *Conopomorpha sinensis*, and *P. operculella* [16,22,23,24,25]. Aromatic and medicinal plants and their essential oils and volatiles are an important source of repellents for the manipulation of phytophagous insects [26,27,28]. Some aromatic plants have demonstrated particularly good results for deterring oviposition, inhibiting fecundity, and lowering the egg-hatching rate [6,24,25,29,30].

*Eryngium foetidum* L. is an ethno-medicinal plant grown in Dehong City, Yunnan Province, China. This plant is widely used in food and medicine due to its flavor and antioxidant capacity, as well as its anti-microbial and anti-inflammatory effects [31,32,33,34]. Extracts of *E. foetidum* have demonstrated a significant anthelmintic effect on *Strongyloides stercoralis* [35]. Additionally, our earlier studies indicated that the original plant material of *E. foetidum* can prevent the PTM from laying eggs on potato tubers (unpublished). However, the volatile compounds of *E. foetidum* responsible for its repellent effect on the PTM are unknown. Thus, in this study, the effects of minced leaves and extracts of *E. foetidum* on the oviposition performance of the PTM were evaluated. The electrophysiologically active compounds of the extracts were screened using chemical analysis and electroantennographic tests. The effects of the EAD-active compounds and their combination on oviposition by PTM females were investigated to identify prospective repellent compounds for the control of this pest species.

## 2. Materials and Methods

### 2.1. Insects

PTMs were collected from a potato field in Dehong, Yunnan Province, China (E 97°58′, N 24°47′), and were reared in the laboratory as described by Gui and Li and Ma and Xiao [21,36]. The larvae were reared on potato tubers, and the adults were fed a 10% honey solution from cotton wicks. The rearing room was kept at a temperature of 24 ± 2 °C, with a photoperiod of 14 h:10 h (light:dark) and 60 ± 5% relative humidity (RH). The pupae were collected and isolated individually in glass vials (8 × 2 cm), after which they were covered with gauze. After emergence, a single female and male were placed in a glass vial for mating. Mated 2-day-old female moths were selected for subsequent experiments. After testing, the females were dissected to detect the presence of spermatophores to confirm mating status.

### 2.2. Preparation of Plant Minced Leaves, Extraction, and Chemicals

#### 2.2.1. Minced Leaves

*E. foetidum* was obtained from a market in Dehong City, Yunnan Province. The fresh plant leaves were washed in tap water and minced into 1-mm-wide sections. The minced leaves were prepared 1 h before the experiment.

#### 2.2.2. Plant Extracts

The fresh minced leaves described above were extracted with dichloromethane at a ratio of 3:10 (weight:volume, g:mL) in ultrasonic waves (53 kHz) for 10 min. The extract was then filtered once, dried using anhydrous sodium sulfate (1 g), filtered through filter paper, and concentrated to an equivalent of 40 g of fresh plant leaves per 1 mL (40 g/mL) using rotary evaporators. Extracts were stored in a refrigerator at 4 °C until use for bioassays and chemical identification. Test solutions at concentrations of 0.156 g/mL, 0.625 g/mL, 2.5 g/mL, and 10 g/mL were diluted in dichloromethane for subsequent experiments. Each solution was prepared 1 h before the relevant experiment.

#### 2.2.3. Chemicals

Dichloromethane, anhydrous sodium sulfate, *trans*-2-dodecenal and *trans*-2-tridecenal were commercial grade (Table 1). *trans*-2-Dodecenal and *trans*-2-tridecenal were diluted in dichloromethane.

### 2.3. Effect of E. foetidum Minced Leaves on Oviposition of PTM in Non-Choice Assay

Fresh minced leaves of *E. foetidum* were placed in the center of the bottom of a mesh cage (35 cm × 35 cm × 35 cm), and then two potato tubers (300 ± 2 g) were placed on the leaves as treatments. Tubers without fresh minced leaves were used as the control treatment. Five mated 2-day-old female moths were transferred into the cage and provided with 10% honey water. The cages of five treatments (6.25, 12.5. 25, 50, and 100 g fresh minced leaves) and one control (0 g fresh minced leaves) were placed at random locations in the laboratory under the same ambient conditions. The numbers of eggs laid on the tubers were counted and recorded after 48 h. Each experiment was repeated four times.

### 2.4. Effect of E. foetidum Minced Leaves on Oviposition of PTM in Choice Assays

Fresh minced leaves of *E. foetidum* were placed on one side of the bottom of a mesh cage (35 cm × 35 cm × 35 cm), and two potato tubers (300 ± 2 g) were placed on the leaves as the treatment. Tubers without fresh minced leaves (control) were placed on the opposite side of the cage, 20 cm apart. Five mated 2-day-old female moths were placed in the cage and provided with 10% honey water. The placement of the cages, the environmental conditions, and the recording methods were the same as in the non-choice experiment of minced leaves. Each experiment was repeated four times.

### 2.5. Effect of E. foetidum Extracts on Oviposition of PTM in Non-Choice Assays

To test the effects of *E. foetidum* extracts on oviposition preference in a non-choice experiment, two potato tubers (300 ± 2 g) were immersed in the extracts at different concentrations or an equivalent volume of solvent (control) for 10 s and then air-dried for 30 min. Five treatments (0.156 g/mL, 0.625 g/mL, 2.5 g/mL, 10 g/mL, and 40 g/mL) and one control (0 g/mL) were transferred into the center of a separate mesh cage (35 cm × 35 cm × 35 cm). Five mated 2-day-old female moths were transferred into the cage and provided with 10% honey water. The cages were placed at random in the laboratory under the same ambient conditions. The placement of cages, the environmental conditions, and the recording method were the same as in the non-choice assay with *E. foetidum* minced leaves. Each experiment was repeated four times.

### 2.6. Effect of E. foetidum Extracts on Oviposition of PTM in Choice Assays

This experiment refers to the oviposition bioassay methods described by Ma and Xiao (2013) with several modifications [21]. The treatment and control tubers were as described above for the non-choice assay of *E. foetidum* extracts. The treatment and control tubers were placed 20 cm apart in a mesh cage. Five mated 2-day-old female moths were placed in the cage and provided with 10% honey water. The placement of the cages, the environmental conditions, and the recording method were the same as in the non-choice experiment of *E. foetidum* minced leaves. Each experiment was repeated four times.

The effects of *E. foetidum* extracts (at the concentrations of 0.156 g/mL, 0.625 g/mL, 2.5 g/mL, 10 mg/mL, and 40 g/mL) on oviposition preference of the PTM females were tested using the above experimental methods.

### 2.7. Coupled Gas Chromatography–Electroantennographic Detection (GC–EAD) Recording

The antennal active compounds of *E. foetidum* extracts were identified using GC-EAD, following the method described by Zhang [5]. A GC (6890A GC, Agilent, Santa Clara, CA, USA) was equipped with an HP-5 capillary column (30 m × 0.32 mm × 0.25 μm, Agilent, Santa Clara, CA, USA) and interfaced with an electroantennographic detector (Syntech, Preetz, Germany). High-purity nitrogen at a flow rate of 25 mL/min was used as the carrier gas. A sample (2 µL) was introduced into the GC column using a split/splitless injector at 250 °C with a flame ionization detector (FID) at 260 °C. The oven temperature was set with programmed increases from 40 °C to 80 °C at a rate of 3 °C/min and was then increased to 280 °C at 5 °C/min, and held for 30 min. The outlet of the GC column effluent was split at a 1:1 ratio, with one part going to the FID of the GC and the other to an excised PTM’s antenna using a Micro fluids Splitter with Makeup Gas (Agilent, Santa Clara, CA, USA).

The antennae for EAD were separated from the head of an active female moth, with the base and distal ends of each antenna cut with a scalpel. The excised antennae were mounted between two microelectrodes using electrode gels. A forked electrode was connected to the EAG probe. The mounted antennae were placed 0.5 cm from the end of the glass tube. A flow of charcoal-filtered and humidified air (850 mL/min) was delivered to the antennae and controlled using a stimulus flow controller (CS-55, Syntech, Preetz, Germany). The signals from the antennae generated by the EAD and FID were amplified 10 times with a high-impedance amplifier (IDAC-2, Syntech, Preetz, Germany) and analyzed with the GC-EAD 2012 software (version 4.6, Syntech, Preetz, Germany). Four GC-EAD recordings with different female antennae were performed. Each antenna was used only once. FID peaks that elicited EAD responses for at least three runs were considered electrophysiologically active and marked for identification using coupled gas chromatography–mass spectrometry (GC-MS, Agilent, Santa Clara, CA, USA).

### 2.8. Chemical Analyses

Samples (2 µL) of *E. Foetidum* extract were analyzed using GC-MS (6890 GC and 5973 MS, Agilent, Santa Clara, CA, USA) as described by Zhang [5]. The GC was equipped with an HP-5 capillary column (30 m × 0.32 mm × 0.25 μm). Helium (1.0 mL/min) was used as the carrier gas. The GC temperature program was the same used for the GC-EAD analyses. Both the injector and transfer line temperatures were 250 °C. Mass spectra were obtained using the electron impact at 70 eV and 230 °C. Data collection and analysis were performed using GC-MS ChemStation software with the Wiley 7n.l mass spectra library (version 7n. l, Wiley Registry of Mass Spectral Data; John Wiley & Sons, Inc., Hoboken, NJ, USA) and the NIST98 database. The compounds were verified by the retention times of the respective standards, and the compounds (including two EAD-active compounds) were quantified using relative area percentage.

### 2.9. Effect of Individual EAD Active Compounds on Oviposition of PTM in Choice Assays

This experimental method was the same as for the *E. foetidum* extract choice assay. The treatment and control tubers were the same as described for the non-choice assay of *E. foetidum* extracts. Five treatments (at the concentrations of 0.625 mg/mL, 1.25 mg/mL, 2.5 mg/mL, 5.0 mg/mL, and 10.0 mg/mL) of each standard EAD-active compound were used, with an equal volume of dichloromethane as a control. The placement of the cages, the environmental conditions, and the recording method were the same as in the non-choice experiment of *E. foetidum* minced leaves. Each experiment was repeated four times.

The effects of two individual standard EAD-active compounds on the oviposition preference of the PTM females were tested using the above experimental methods.

### 2.10. Effect of Mixtures of EAD Active Compounds on Oviposition Preference of PTM

A mixture (50 mL) of the two EAD-active compounds at their natural ratio (7.22:1) (10 mg/mL as Blend 1, and 5 mg/mL as Blend 2) was used as a treatment for the assays, with an equal volume of dichloromethane as a control. The effects of the different mixtures of GC-EAD active compounds on the oviposition selection of the PTM were tested in choice experiments under the same conditions as in the *E. foetidum* extract choice assay. The placement of the cages, the environmental conditions, and the recording method were the same as used for the non-choice experiment with *E. foetidum* minced leaves. Each experiment was repeated four times.

### 2.11. Statistical Analysis

All data were analyzed using SPSS16.0 (IBM Corporation, Armonk, NY, USA). The differences in the number of eggs between the treatment and control groups in the non-choice experiment were tested using one-way ANOVA, with comparisons performed using Tukey’s multiple comparison test (*p* < 0.05). The differences in the number of eggs between the treatment and control groups in the choice experiment were analyzed with a Chi-square test at the level of *p* < 0.01. The attraction or deterrent effects of *E. foetidum* minced leaves, *E. foetidum* extracts, and EAD-active compounds and their mixtures on the oviposition performance of mated females in the choice experiment were tested using the oviposition stimulation index (OSI) [37], combined with a Chi-square test. The OSI was calculated as follows:

OSI = (eggs in the treatment group − eggs in the control group) × 100/(eggs in the treatment group − eggs in the control group). An OSI of <0 indicated that the tested material had a deterrent effect on oviposition, and an OSI of >0 indicated that the tested material had a promoting effect.

## 3. Results

### 3.1. Effects of E. foetidum Minced Leaves on Oviposition Preferences of P. operculella in Non-Choice Assays

**Figure 1:**
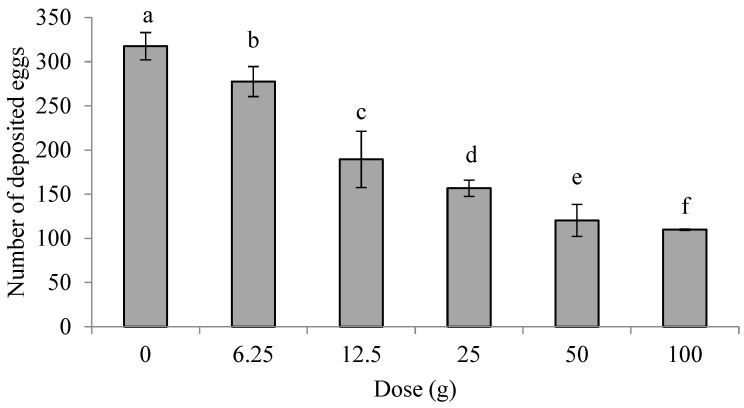
Effects of minced leaves of *E. foetidum* on oviposition preferences of *P. operculella* in non-choice experiment. Lowercase letters indicate significant differences between concentrations (ANOVA followed by Tukey’s multiple comparison test) (*p* < 0.05).

The number of eggs deposited in the treatment groups at doses from 6.25 g to 100 g was significantly lower than that in the control group(*p* < 0.05) (Figure 1). The results indicate that the minced leaves of *E. foetidum* had a deterrent effect on oviposition by PTM females at the tested doses.

### 3.2. Effects of Minced Leaves of E. foetidum on Oviposition Preferences of P. operculella in Choice Assays

**Figure 2:**
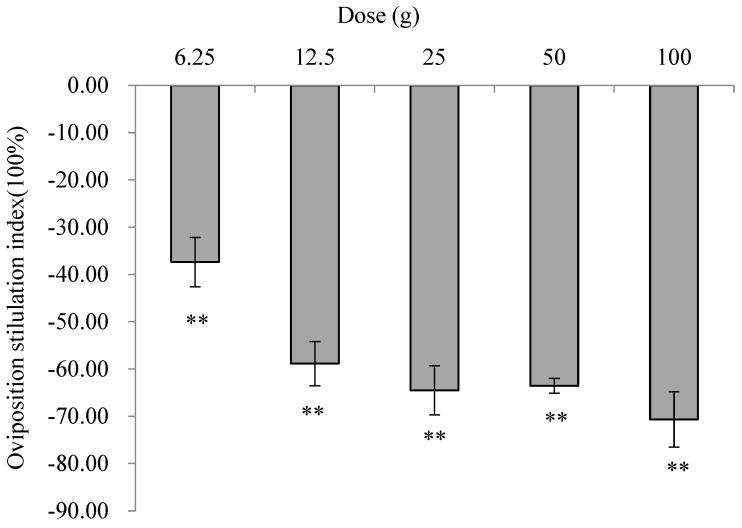
Effects of minced leaves of *E. foetidum* on oviposition preferences of *P. operculella* in choice experiments. A double asterisk (**) indicates significant differences between the number of eggs oviposited on the treatment and control sides using χ^2^ tests (*p* < 0.01).

The number of eggs deposited on tubers treated with minced leaves of *E. foetidum* at doses of 6.25–100 g was significantly lower than that in the control treatment. All OSI values were less than 0 (6.25 g: χ^2^ = 27.77, *p* < 0.001; 12.5 g: χ^2^ = 61.39, *p* < 0.001; 25 g: χ^2^ = 107.58, *p* < 0.001; 50 g: χ^2^ = 99.86, *p* < 0.001; and 100 g: χ^2^ =127.48, *p* < 0.001), showing a significant repellent effect on oviposition. All OSIs decreased with increasing dosages, with a minimum of −70.68% at 100 g (Figure 2).

### 3.3. Effects of E. foetidum Extracts on the Oviposition Preferences of P. operculella in Non-Choice Assays

**Figure 3:**
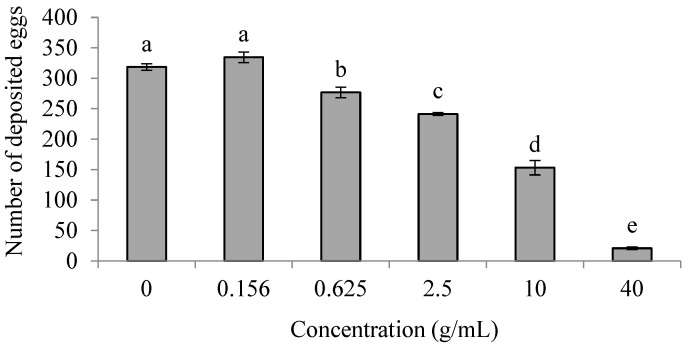
Effects of *E. foetidum* extracts at different concentrations on PTM oviposition preference in non-choice experiment. Lowercase letters indicate significant differences between concentrations (ANOVA followed by Tukey’s multiple comparison test) (*p* < 0.05).

The number of eggs in the treatment groups at a concentration of 0.156 g/mL did not significantly differ from the control, and the numbers of eggs in the treatment groups at concentrations from 0.625 g/mL to 40 g/mL were significantly lower than in the control treatment (*p* < 0.05) (Figure 3). The results indicate that the *E. foetidum* extracts had a deterrent effect on the oviposition of PTM females at concentrations ranging from 0.625 g/mL to 40 g/mL.

### 3.4. Effects of E. foetidum Extracts on Oviposition Preferences of P. operculella in Choice Assays

**Figure 4:**
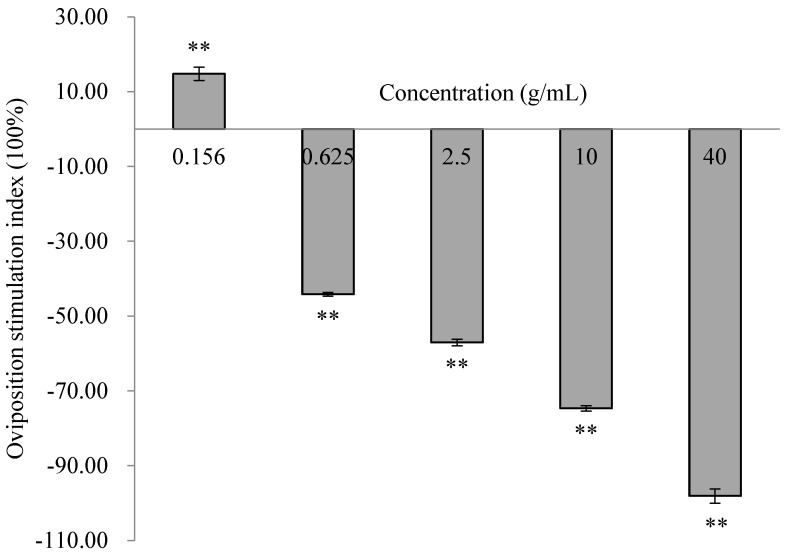
Effect of *E. foetidum* extracts at different concentrations on oviposition preference in choice experiments. A double asterisk (**) indicates significant differences between oviposition on the treated and control sides using χ^2^ tests (*p* < 0.01).

The number of eggs laid on the treated tubers at a concentration of 0.156 g/mL of *E. foetidum* extract was significantly greater than on the control tubers. OSI > 0 indicated that the extract had an attractive effect at this dosage (χ^2^ = 8.63, *p* < 0.01). Significantly fewer eggs were deposited on the tubers treated with the extracts at concentrations of 0.625 g/mL to 40 g/mL than on the control. All OSIs for 0.625 g/mL to 40 g/mL were less than 0 (0.625 g/mL: χ^2^ = 64.89, *p* < 0.001; 2.5 g/mL: χ^2^ = 121.63, *p* < 0.001; 10 g/mL: χ^2^ = 215.82, *p* < 0.001; and 40 g/mL: χ^2^ = 310.11, *p* < 0.001), indicating a significant repellent effect on oviposition. All OSIs decreased with increasing concentrations, with a minimum of −98.10% at 40 g/mL (Figure 4).

### 3.5. Chemical and Electrophysiological Analyses

**Figure 5:**
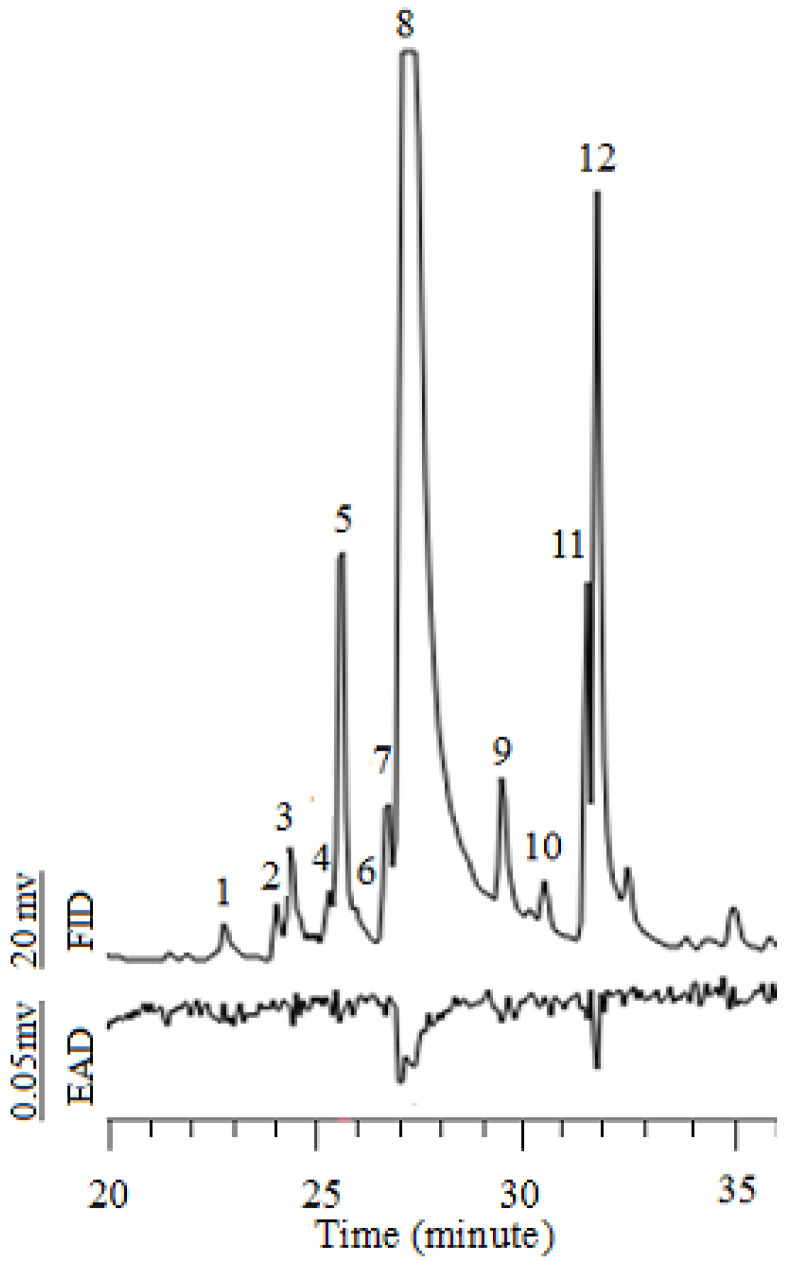
GC-EAD responses of PTM to *E. foetidum* extract (N = 4). 1: Undecanal; 2: 2,4,5-Trimethylbenzaldehyde; 3: 2-Undecenal; 4: *trans*-4-Nonenal; 5: Dodecanal; 6: *trans*-Caryophyllene; 7: 2-Dodecenal; 8: *trans*-2-Dodecenal; 9: 3-Dodecenal; 10: Tetradecanal; 11: Unknown; 12: *trans*-2-Tridecenal.

GC-MS and GC-EAD analyses were performed to detect the electrophysiological responses in the antennae of mated PTM females to the *E. foetidum* extracts to screen and identify the EAD-active components of the PTM. Eleven compounds were identified from twelve compounds in the *E. foetidum* extracts. The relative quantities were determined using GC-FID (Table 2). *trans*-2-Dodecenal and *trans*-2-tridecenal were the dominant compounds, with relative proportions of 68.39% and 9.46%, respectively. *trans*-2-Dodecenal and *trans*-2-tridecenal were the active compounds that elicited antennal responses in the mated females by comparing the GC-EAD and GC-MS spectrograms (Figure S1 and Figure 5).

### 3.6. Oviposition Responses of PTM Females to Individual EAD Active Compounds

**Figure 6:**
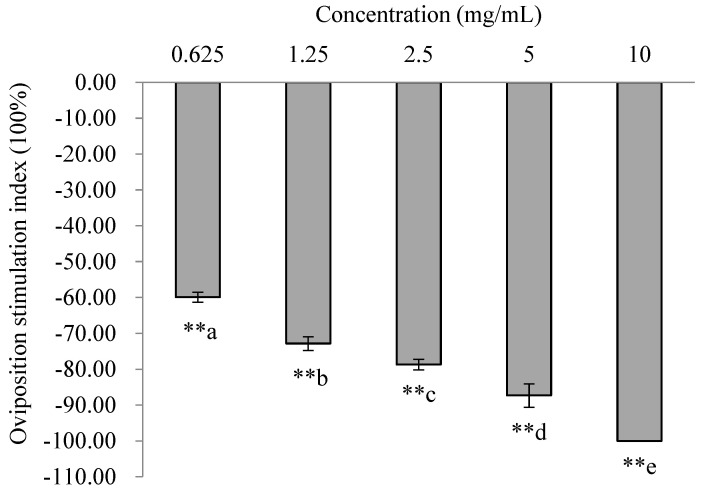
Oviposition responses of PTM females to *trans*-2-dodecenal at different concentrations. A double asterisk (**) indicates significant differences between oviposition on the treated and control sides using χ^2^ tests (*p* < 0.01). Lowercase letters indicate significant differences between concentrations (ANOVA followed by Tukey’s multiple comparison test) (*p* < 0.05).

All the OSIs of *trans*-2-dodecenal were <0, and the number of eggs laid on the treatment was significantly lower than that on the control. Thus, *trans*-2-dodecenal showed an extremely significant repellent effect on oviposition at the tested concentrations (0.62 mg/mL: χ^2^ = 98.88, *p* < 0.001; 1.25 mg/mL: χ^2^ = 137.92, *p* < 0.001; 2.5 mg/mL: χ^2^ = 184.70, *p* < 0.001; 5 mg/mL: χ^2^ = 204.86, *p* < 0.001; and 10 mg/mL: χ^2^ = 256.29, *p* < 0.001). The OSIs significantly decreased with increasing concentrations, indicating that the repellent effect increased with increasing concentration. The OSI at 10 mg/mL was extremely low, reaching −100% (Figure 6).

**Figure 7:**
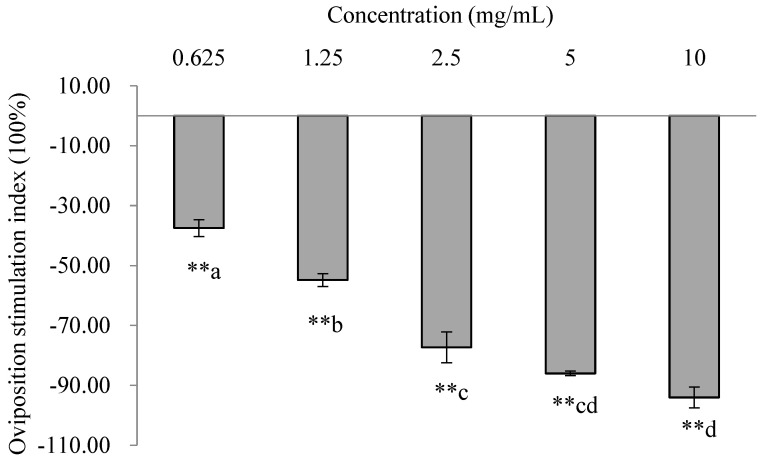
Oviposition responses of PTM females to *trans*-2-tridecenal at different concentrations. A double asterisk (**) indicates significant differences between oviposition on the treated and control sides using χ^2^ tests (*p* < 0.01). Lowercase letters indicate significant differences between concentrations (ANOVA followed by Tukey’s multiple comparison test) (*p* < 0.05).

All the OSIs of *trans*-2-tridecenal were < 0, and significantly fewer eggs were laid on the treatment than on the control. Thus, *trans*-2-tridecenal had an extremely significant repellent effect on oviposition at the tested concentrations (0.625 mg/mL: χ^2^ = 35.96, *p* < 0.001; 1.25 mg/mL: χ^2^ = 42.05, *p* < 0.001; 2.5 mg/mL: χ^2^ = 100.36, *p* < 0.001; 5 mg/mL: χ^2^ = 110.21, *p* < 0.001; and 10 mg/mL: χ^2^ = 139.64, *p* < 0.001). The OSIs significantly decreased with increasing concentration, showing that the repellent effect increased with increasing concentration. The concentration of 10 mg/mL had the strongest repellent effect at 94.03% (Figure 7).

### 3.7. Oviposition Responses of PTM Females to Mixtures of EAD Active Compounds

**Figure 8:**
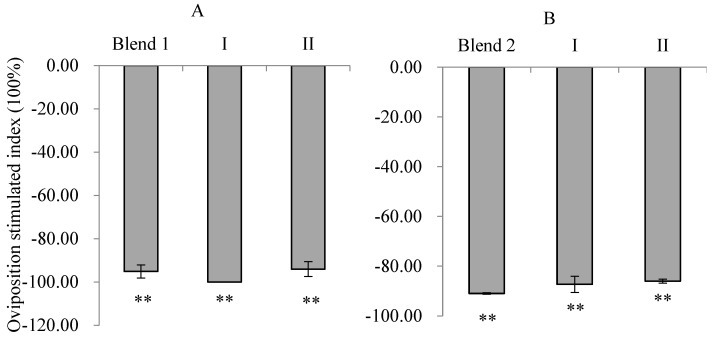
Oviposition responses of PTM females to mixtures of *trans*-2-dodecenal and *trans*-2-tridecenal at different concentrations. (**A**): 10 mg/mL; (**B**): 5 mg/mL. I: *trans*-2-dodecenal; II: *trans*-2-tridecenal. Blend: a mixture of the two EAD active compounds of 10 mg/mL (Blend 1) and 5 mg/mL (Blend 2) at their natural ratio. A double asterisk (**) indicates significant differences between oviposition on the treated and control sides using χ^2^ tests (*p* < 0.01).

The OSI value for Blend 1 was −95.11%, indicating an extremely significant repellent effect on oviposition (χ^2^ = 142.41, *p* < 0.001). The repellent effect of Blend 1 was stronger than that of *trans*-2-tridecenal (−94.0%) but weaker than that of *trans*-2-dodecenal (−100.0%). There was no significant difference between the two (*p* < 0.05) (Figure 8A).

The OSI for Blend 2 was −90.96%, indicating an extremely significant repellent effect on oviposition (χ^2^ = 109.09, *p* < 0.001). Blend 2 had a synergistic repellent effect compared with the single components of *trans*-2-dodecenal (−87.32%) and *trans*-2-tridecenal (−86.02%) at a concentration of 5 mg/mL, but there was no significant difference (*p* < 0.05) (Figure 8B).

## 4. Discussion

The results of this study showed that the *E. foetidum* extracts had significant repellent effects on the oviposition ofthe PTM. This is consistent with previous research reporting that compounds from non-host plants, especially aromatic plants, can deter oviposition by the PTM [30,38]. The dried powders of the aromatic plants *Curauma longa* [24], *Allium cepa* [24], and *Ocimum basilicum* [6,24], and oils of *Cymbopogon citrates* [29], *Myristica fragrans* [29], *Mentha citrate* [29] and *Lindera glauca* [39] had significant inhibition and repellent effects on PTM oviposition. Further research should be carried out to evaluate the effect of *E. foetidum* dried powder or oil on PTM oviposition.

In addition to oviposition deterrence, studies showed that the essential oils of the aromatic plants *Cinnamomum zeylanicum* [40], *Rosmarinus officinalis* [30,41] *C. citrates* [29], and *M. fragrans* [29] had a fumigation toxicity effect on larval and adult PTMs. *Coriandrum sativum*, *Zygophyllum coccineum* and *O. basilicum* significantly inhibited the growth and development of larvae of *P. operculella* [42]. The methanol crude extract of *Lantana camara* flowers and *Solanum nigrum* fruits prevented feeding on *P. operculella* [43]. Further investigations of fumigation activity, inhibiting fecundity, and anti-feeding effects should be conducted to evaluate the potential control efficacy of *E. foetidum* against the PTM.

The analysis revealed that the volatile compounds in *E. foetidum* extracts included undecanal, 2, 4, 5-trimethylbenzaldehyde, dodecanal, *β*-caryophyllene, 2-dodecenal, *trans*-2-tridecenal, and tetradecanal, consistent with Paul’s findings [33]. Additionally, *trans*-4-nonenal, 2-undecenal, *trans*-2-tridecenal, tetradecanal, *cis*-2-dodecenal, *cis*-2-tridecenal and dodecanol were identified in the essential oil and volatile components of *E. foetidum* in a study conducted by Acharya, Chowdhury and Quynh [44,45,46]. These results aligned with previous findings indicating *trans*-2-dodecenal as the primary volatile component in *E. foetidum* [45,46,47].

This work indicated that both *trans*-2-dodecenal and *trans*-2-tridecenal had significant repellent effects on oviposition by PTM females, and *trans*-2-dodecenal at 10 mg/mL achieved a 100% repellent effect. This observation was similar to previously reported results showing that *E. foetidum* extracts rich in *trans*-2-dodecenal had remarkably anthelmintic effects against *S. stercoralis* [35].The combined effect of *trans*-2-dodecenal and *trans*-2-tridecenal exceeded that of the individual compounds at the concentration of 5 mg/mL, aligning with the reported repellent effect of a dipentene and (±)-citronellal mixture against female adults of *Drosophila melanogaster* [48]. Therefore, *trans*-2-dodecenal, *trans*-2-tridecenal and their mixture could be developed into oviposition repellents to prevent damage by the PTM.

Several studies have suggested that aldehydes such as heptanal, octanal, hexanal, and nonanal can inhibit oviposition by the PTM [21,49,50]. In this study, *trans*-2-dodecenal and *trans*-2-tridecenal also showed repellent effects against the PTM; thus, aldehydes may be one class of oviposition-repellent compounds against the PTM. Previous studies have screened some oviposition-repellent substances from frass and plants. Further studies could investigate the impact of binary and multiple components on deterring oviposition by the PTM to optimize repellent strategies [5,24,25,29].

Because plant volatiles and essential oils have the disadvantages of strong volatility and short persistence duration, a method of improving their persistence effect needs to be applied. Previous studies showed that the fumigation time and toxicity of *C. zeylanicum* essential oil adsorbed on nanomaterials was enhanced due to the slow-release effect of the nanomaterials [40]. In the future, we can combine repellent compound ingredients with nanomaterials to extend their persistence periods. In addition, laboratory experiments differ from real field environments and storage warehouse conditions, so further bioassays of oviposition repellents in fields and storage warehouses must be carried out to verify the usability and practical doses of these compounds in future applications and product development.

Previous research has demonstrated the effectiveness of push–pull strategies in pest control [21,51]. Some attractants have been screened from plants, so potatoes or attractant-treated potatoes can be used as trap sources [21,36,39,49,52]. Considering the abundant resources and low price of *E. foetidum*, in storage, we can directly put fresh plants, dry powder and extracts of *E. foetidum* on potatoes as a repellent source. Additionally, *E. foetidum* volatile compounds and essential oil, alone or absorbed on nanomaterial-treated potatoes, can be used as a push treatment in storage and fields. We should investigate the push–pull dynamic effects of different combinations of push/repellent treatments and pull/attractant treatments. In the field, we can also use *E. foetidum* and potato intercropping to deter PTM oviposition as a repellent, using the attractant combined with the trap as a trap source. Field and storage trials are essential to assess the regulatory impact of different combinations on PTM oviposition. Such studies will provide valuable insights for devising effective control strategies for managing *P.operculella* damage.

Most plant oils play a key role in stored grain protection, which can reduce the need for the use of insecticides and retard the development of pest resistance. The repellent bioactive compounds derived from the aromatic and medicinal plant *E. foetidum* are relatively safe, low-residue and eco-friendly for controlling the PTM. Therefore, *E. foetidum* may be a potential alternative material to synthetic insecticides for the control of *P. operculella.*

## 5. Conclusions

The results of this research demonstrated that *E. foetidum* leaves and extracts possessed repellent effects on oviposition by PTM adult females, and the repellent effect increased with the concentration in non-choice and choice experiments. *trans*-2-dodecenal and *trans*-2-tridecenal were the main components in *E. foetidum* extracts and elicited antennal responses in PTM females. The EAD-active compounds, individually and in mixtures, had significant oviposition-repellent effects against the PTM. Therefore, *trans*-2-dodecenal and *trans*-2-tridecenal may have potential applications as oviposition deterrents in the control of the PTM.

## Figures and Tables

**Table 1:** Purity and source of standard chemical compounds.

Compound	CAS	Purity (%)	Source
Dichloromethane	75-09-2	Analytically pure (99.9%)	Tianjin Reagent Co., Ltd., Tianjin, China
Anhydrous sodium sulfate	7757-82-6	Analytically pure (99.9%)	Tianjin Reagent Co., Ltd., Tianjin, China
*trans*-2-Dodecenal	20407-84-5	≥90.0	TCI, Tokyo, Japan
*trans*-2-Tridecenal	7069-41-2	≥96.0	Sigma-Aldrich, Shanghai, China

**Table 2:** Compounds identified in the *E. foetidum* extracts.

Number	Compound	RT (Min)	Relative Area (%)	GC-EAD Active or Not
1	Undecanal	22.87	0.21	
2	2,4,5-Trimethylbenzaldehyde	24.33	0.15	
3	2-Undecenal	24.44	0.65	
4	*trans*-4-Nonenal	25.36	0.47	
5	Dodecanal	25.60	3.88	
6	*trans*-Caryophyllene	25.93	0.20	
7	2-Dodecenal	26.72	1.96	
8	*trans*-2-Dodecenal	27.22	68.39	※
9	3-Dodecenal	29.62	1.31	
10	Tetradecanal	30.63	0.50	
11	Unknown	31.77	3.87	
12	*trans*-2-Tridecenal	31.97	9.46	※

The symbol “※” means that this compound elicited GC-EAD responses in the PTM.

## Data Availability

The data will be made available upon request.

## Supplementary Materials

The following supporting information can be downloaded at: https://www.mdpi.com/article/10.3390/insects16020158/s1, Figure S1: Full scale of GC-EAD responses of the PTM to the *E. foetidum* extract.
