# Supplementary figures and images for: Oviposition Deterrents from Extracts of Eryngium foetidum Against Potato Tuber Moth Phthorimaea operculella Zeller (Lepidoptera: Gelechiidae)
Source: Insects. 2025 Feb 4;16(2):158. doi: 10.3390/insects16020158 (PMC11855822; doi:10.3390/insects16020158)

## GC-EAD

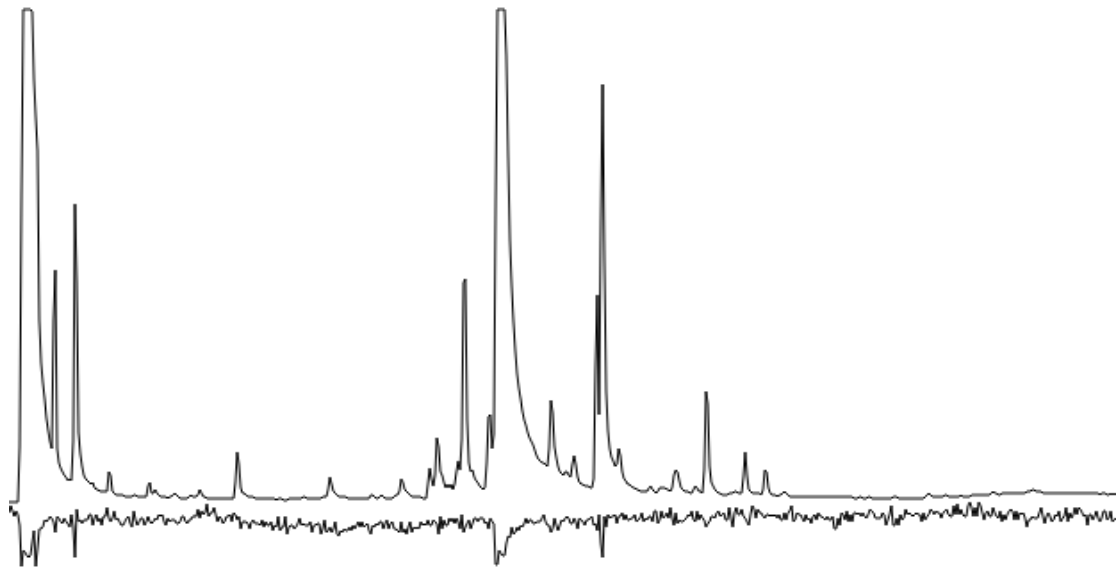

Full scale of GC-EAD responses of the PTM to the *E. foetidum* extract

Supplement: Supplementary file 1 [file insects-16-00158-s001.zip › insects-3428840-supplementary.pdf]
